# Carbonic anhydrases III and IX are new players in the crosstalk between adrenocortical carcinoma and its altered adipose microenvironment

**DOI:** 10.1007/s40618-023-02008-4

**Published:** 2023-01-16

**Authors:** L. Fei, G. Cantini, A. Nocentini, P. Nardini, S. Catarinicchia, L. Canu, T. Ercolino, G. Quartararo, G. Nesi, M. Gacci, M. Maggi, C. Hantel, M. Mannelli, C. T. Supuran, M. Luconi

**Affiliations:** 1 Endocrinology Unit, Department of Experimental and Clinical Biomedical Sciences “Mario Serio”, University of Florence, 50139 Florence, Italy; 2 Centro di Ricerca e Innovazione sulle Patologie Surrenaliche, AOU Careggi, 50134 Florence, Italy; 3 ENS@T Center of Excellence, Florence, Italy; 4 Pharmaceutical and Nutraceutical Section, Neurofarba Department, University of Florence, Sesto Fiorentino, 50019 Florence, Italy; 5 Platform of Imaging, Department of Experimental and Clinical Medicine, University of Florence, 50139 Florence, Italy; 6 Endocrinology Unit, Careggi University Hospital (AOUC), 50139 Florence, Italy; 7 General, Bariatric and Metabolic Surgery Unit, Santa Maria Nuova Hospital, Piazza Santa Maria Nuova, 1, 50122 Florence, Italy; 8 Department of Health Sciences, University of Florence, Viale Pieraccini, 6, 50139 Florence, Italy; 9 Department of Minimally Invasive, Robotic Urologic Surgery & Kidney Transplantation, Careggi University Hospital (AOUC), Florence, Italy; 10 Department of Endocrinology, Diabetology and Clinical Nutrition, University Hospital Zurich (USZ), University of Zurich (UZH), CH-8091 Zurich, Switzerland; 11 Medizinische Klinik und Poliklinik III, University Hospital Carl Gustav Carus Dresden, 01307 Dresden, Germany

**Keywords:** Rare cancer, Adipose precursors, Carbonic anhydrases, Tumor microenvironment, Obesity, ACC

## Abstract

**Purpose:**

Adrenocortical carcinoma (ACC), a rare malignancy of the adrenocortex, is characterized by a crosstalk between the adipose microenvironment and tumor. Here, we assessed the involvement of carbonic anhydrase (CA) enzymes III and IX (CAIII and CAIX), in the metabolic alterations of the adipose tissue characterizing obesity and in the local crosstalk between the tumor adipose microenvironment and ACC.

**Results/methods:**

CAIII and CAIX expression is altered in visceral adipose tissue (VAT) in obesity and in ACC. A significant CAIX upregulation was present in ACC at advanced stages (*n* = 14) (fold increase FI = 7.4 ± 0.1, *P* < 0.05) associated with lower CAIII levels (FI = 0.25 ± 0.06, *P* < 0.001), compared with lower stages (*n* = 9). In vitro coculture between visceral adipose stem cells (ASCs) and ACC cell lines, H295R and MUC-1, mimicking the interaction occurring between VAT and advanced ACC, showed a significant CAIX upregulation in H295R but not in MUC-1 cells, and a decreased expression of CAIII. The effect on adipose cells was different when cocultured with H295R or MUC-1 cells. Coculture did not modulate CAIII expression in ASCs, which, however, was significantly downregulated with H295R (FI = 0.34 ± 0.11, *P* < 0.05) and upregulated by MUC-1 when cocultured ASCs were induced to differentiate toward adipocytes, with an expression profile similar to what found in VAT of obese subjects. CAIX expression was markedly increased in ASCs cocultured with H295R and to a less extent following adipogenesis induction (FI = 150.9 ± 46.5 and FI = 4.6 ± 1.1, *P* < 0.01, respectively).

**Conclusion:**

Our findings highlight a modulation of CAIII and CAIX in the metabolic crosstalk between ACC and its local adipose microenvironment, suggesting that CAs might represent a potential target for novel anticancer therapies.

## Introduction

The adipose tissue (AT) is a pivotal endocrine organ for the regulation not only of energetic and metabolic equilibrium, but it is also involved in solid tumor development and progression. A different sensitivity to metabolic dysregulation characterizes the adipose compartments, in particular being visceral fat surrounding most visceral organs including the adrenal gland, more prone than subcutaneous fat to metabolic alterations underlying obesity. The adipose cell is among the main actors of the tumor microenvironment able to control the tumor development and progression [1, 2]. Adrenocortical carcinoma (ACC) is a rare endocrine malignancy affecting the adrenal cortex. In advanced carcinoma, adrenocortical cancer cells infiltrate the visceral fat mass surrounding the adrenal, establishing a crosstalk between the tumor mass and the local adipose microenvironment, and resulting in a reciprocal cell reprogramming characterized by microenvironment acidification and alteration of the cell metabolism [3, 4]. Among the enzymes involved in intracellular and extracellular pH regulation, carbonic anhydrases (CAs) are ubiquitous metalloenzymes that catalyze the conversion of CO_2_ to bicarbonate and protons. In addition, CAs are involved in biosynthetic reactions, such as gluconeogenesis and lipogenesis, as well as they are also associated with tumor metabolism, suggesting that CAs in the tumor and in the adipose microenvironment may represent a potential target for cancer treatment. Among the different isoforms of CAs, carbonic anhydrase III is a cytoplasmic isoenzyme abundantly expressed in metabolically active tissues, as adipose tissue, skeletal muscle, and liver [5, 6]. CAIII is the most abundant enzyme in mature adipocytes, where it might scavenger the high levels of reactive oxygen species (ROS) and lipid peroxidation products generated during lipid metabolism and fatty acid oxidation. Low levels of CAIII characterize the preadipocyte state and are necessary to elicit PPARγ expression, the key early gene controlling the adipogenic process [7]. For its specific involvement in oxidative stress, a pivotal process in supporting tumor evolution, CAIII expression and role in cancer deserve a deeper investigation and have never been assessed in ACC.

CAIX is strongly expressed in many solid tumors, including ACC [8]. Its extracellular catalytic activity maintains a controlled intracellular pH equilibrium in highly active tumor cells by hydrating the CO_2_ diffusing outside resulting in acidification of the extracellular tumor microenvironment [9, 10], being a hallmark of tumor aggressiveness and invasiveness [11]. In addition, CAIX level dramatically increases in response to hypoxia via a direct transcriptional activation of *CA9* gene by hypoxia inducible factor-1 [10, 12]. For its role in pH regulation and in processes characterized by hypoxic condition, it might be of interest to explore CAIX presence in adipose cells and its regulation at the level of the adipose microenvironment characterizing ACC.

Therefore, the aim of the present study was to assess the involvement of CAIII and CAIX in the local crosstalk between the tumor adipose microenvironment and ACC, starting from the evaluation of the reciprocal expression of these enzymes in the metabolic alterations of the adipose tissue characterizing obesity and which may be shared by the adipose niche surrounding ACC.

## Materials and methods

### Adipose tissue and adrenal tissue specimens: patient cohorts and ethical approval

Visceral and subcutaneous abdominal adipose tissue specimens (VAT and SAT) were obtained from *n* = 4 lean patients (50% females, age = 75 ± 16 years, body mass index—BMI = 22.3 ± 1.4 kg/m^2^) undergoing abdominal surgery for cholecystectomy at AOU Careggi University Hospital, and *n* = 8 morbid obese subjects (50% females, age = 61 ± 14 years, BMI = 46.8 ± 14.6 kg/m^2^) undergoing bariatric surgery at Santa Maria Nuova Hospital.

Normal adrenals (*n* = 2) were collected during nephrectomy or following cadaveric explants, while adrenocortical cancer specimens were obtained from *n* = 23 patients affected by ACC and undergoing tumor resection, whose characteristics are summarized in Table 1. The histologic diagnosis of ACC was performed by the referent pathologist (G.N.) on the tumor tissue removed at surgery. The tumor specimens were evaluated according to the Weiss System. The Ki67 proliferation index was assessed using the anti-human Ki67 monoclonal MIB1 antibody (Dako, Carpenteria, CA, USA). Ki67 positive nuclei were counted on 1000 tumor cells and Ki67 LI was expressed as the percentage of proliferating cells. Tumors were staged according to the revised TNM classification of ACC by European Network for the Study of Adrenal Tumors (ENS@T) [13].

**Table 1:** Clinical characteristics of the ACC cohort

ACC patient Cohort (*n* = 23)	
Age (years)	63 ± 12
Male sex (%)	10 (43)
BMI (kg/m^2^)	27 ± 5
Secretion (%)	
Nonfunctional	8 (35)
Glucocorticoids	9 (38)
Sex steroids	4 (17)
Mineralcorticoids	1 (4)
NA	1 (4)
Tumor diameter (cm)	9 ± 5
Ensat stage	
I	6 (26)
II	3 (13)
III	10 (44)
IV	4 (17)
Weiss score	6 ± 2
KI67 LI	22 ± 20

Anthropometric data and clinical features are reported for the cohort of ACC patients. Data are expressed as mean ± SD for parametric continuous variables (age, BMI, tumor diameter, Ki67 LI and Weiss), and as absolute number and percentage (brackets) of patients for the other non-continuous variables (sex, secretion, stage)

*NA* not available, *BMI* body mass index, Ki67 *LI* labeling index

Patients were enrolled and tissue samples were obtained after written informed consent to the study, according to the approval from the local ethical committee (Rif. 58/11 version 1.2 date 05.10.2015, and Rif. 59/11 version 1.3 date 05/04/2019, for adipose tissue and ACC tissue specimens, respectively). Tumor and adipose tissue samples were obtained during surgery, snap-frozen and stored at −80 °C until protein and RNA extraction, or alternatively formalin fixed for immunofluorescence and histochemical analysis.

### Cell cultures

The human adrenocortical carcinoma cell line H295R was obtained from the American Type Culture Collection (ATCC) and cultured in DMEM/F-12 medium with 10% FBS, 2 mM L-glutamine, 100 U/ml penicillin–100 μg/ml streptomycin and a mixture of insulin/transferrin/selenium (ITS) (Sigma-Aldrich).

The MUC-1 cells, kindly provided by Dr. Constanze Hantel, were cultured in Advanced DMEM/F12 medium (Thermo-Fisher) with 10% FBS, 2 mM L-glutamine, and 100 U/ml penicillin–100 μg/ml streptomycin as previously described [14].

Human primary adipose stem cells (ASCs) were isolated from the stromal vascular fraction derived from visceral adipose tissue biopsies as described elsewhere [15, 16]. ASCs were cultured in DMEM with 20% FBS, 2 mM L-glutamine, 100 U/ml penicillin–100 μg/ml streptomycin, and 1 μg/ml amphotericin-B (Sigma-Aldrich).

All cells were incubated at 37 ^◦^C in a humidified 5% CO_2_ atmosphere.

ASCs plated in six-well plates were induced to differentiate toward mature adipocytes (Adipo) for 15 days with DIM cocktail (DMEM without phenol red with 10% FBS plus 0.5 mM 3-isobutyl-1-methylxanthine, 1 µM dexamethasone, 10 µM insulin, and 1 μM rosiglitazone).

### Coculture experiments

In the coculture experiments, we used ThinCert™ tissue culture insert for six-well plate with 0.4 µm pore size (Greiner Bio-One, Kremsmünster, Austria), as previously described [3]. Briefly, ASCs and cancer cells were seeded in six-well plates or in cell culture insert, respectively (ASCs, 5 × 10^4^ cells/well, H295R, 10^5 ^cells/insert and MUC-1, 3 × 10^4^ cells/insert), unless otherwise indicated, with their own complete medium for 2 days before the coculture experiments. At the beginning of coculture, inserts containing H295R/MUC-1 were transferred to the top of the wells seeded with ASCs, and all cells were grown in DMEM plus 10% FBS.ASCs and H295R or MUC-1 cells plated alone at the same density as monoculture were used as control monolayers. For coculture experiments between H295R or MUC-1 cells and mature adipocytes (Adipo), ASCs were seeded in six-well plates (5 × 10^4^ cells/well) and cultured alone or with H295R or MUC-1 in the presence of DIM cocktail for 10 days to obtain in vitro adipose-derived adipocytes [3]. In parallel, ASCs cultured only in 10% FBS-DMEM (i.e., not adipogenic medium) for the same time interval, were used as a negative control.

### Immunohistochemistry analysis of adipose tissue and ACC specimens

Samples were fixed in 4% paraformaldehyde/ 0.1 m phosphate buffered saline (PBS) and washed with PBS for 1 h at room temperature (RT). After gradient dehydration and paraffin embedding, blocks were cut into 5 μm sections. Sequential full-thickness cross-sections of AT, ACC and normal adrenal samples were stained with H&E or processed for immunofluorescence. For immunofluorescence staining, deparaffinized and rehydrated slices were treated for antigen retrieval for 10 min at 100 °C in Tris buffer (10 mM), followed by cooling to RT. The sections were then washed in PBS, blocked with 1.5% bovine serum albumin (BSA, Applichem, Darmastad, Germany) in PBS for 20 min at RT to minimize non-specific binding. The primary antibodies (CAIII, 1:100 sc-373729 and CAIX, 1:100 sc-365900, Santa Cruz Biotechnology, Santa Cruz, CA) were diluted in BSA 1.5% PBS and were incubated overnight at 4 °C. The omission of the primary antibodies was used as negative control. The next day, the sections were incubated for 2 h at RT in the dark with appropriate fluorochrome-conjugated secondary antibodies diluted in BSA 1.5% PBS (Alexa Fluor anti-mouse 594/488, Jackson ImmunoResearch Labs, West Grove, PA, USA). Subsequently, the specimens were rinsed three times with PBS and then mounted with DAPI aqueous medium (Fluoroshield™ with DAPI, Thermo Fisher Scientific). The immunolabeled sections were observed under an epi-fluorescence Olympus BX40 microscope coupled to analySIS∧B Imaging Software (Olympus, Milan, Italy) equipped with 20 × and 40 × objectives.

### mRNA isolation and quantitative real-time RT-PCR

mRNA isolated from tumor and adipose tissues or from cell cultures as detailed previously [16, 17] were subjected to quantitative real-time RT-PCR (qRT-PCR) for the following genes: *CA3*, *CA9*, *ADIPOQ* (adiponectin)*, FABP4* (fatty acid binding protein 4) and *GAPDH* genes (Taqman Gene Expression Assay, Life Technologies, respective codes: Hs00193123_m1, Hs00154208_m1, Hs00605917_m1, Hs00609791_m1, FAM-MGB 4325934–1301038). The amount of target, normalized to the endogenous reference gene (*GAPDH*) and relative to a calibrator (Stratagene, La Jolla, CA, USA), was calculated by 2^−ΔΔCt^.

### Sodium dodecyl sulfate–polyacrylamide gel electrophoresis (SDS-PAGE) and Western blot analysis

Tumor and adipose tissue lysates were obtained as previously detailed [16, 17]. Tissue/cell proteins were extracted in RIPA buffer (20 mM Tris, pH 7.4, 150 mM NaCl, 0.5% Triton-100, 1 mM Na_3_VO_4_, 1 mM PMSF). Thirty micrograms of proteins were separated by reducing SDS-PAGE stain-free precast gels^®^ (Bio-Rad) and transferred to polyvinylidene difluoride (PVDF) membranes (Trans-Blot^®^ TurboTM, Bio-Rad). Membranes were probed with the following primary antibodies: CAIII (1:500, sc-373729, Santa Cruz Biotechnology, Santa Cruz, CA) and CAIX (1:500 sc-365900, Santa Cruz Biotechnology, Santa Cruz, CA). Each membrane was incubated overnight at 4 °C with primary antibodies followed by peroxidase-conjugated secondary IgGs (1:3000). Image acquisition and analysis were performed with Image Lab software version 6.0 on a ChemiDoc TM Touch instrument (Bio-Rad), using fluorescence emission of protein bands separated on stain-free gels for the total lane normalization [18].

### Statistical analysis

Statistical analysis was performed using SPSS software 27.0 (Statistical Package for the Social Sciences, Chicago, US) for Windows. The Kolmogorov–Smirnov’s test was used to assess normal distribution of data. Results are expressed as mean ± SE, unless otherwise stated. Student’s *t* test was used for comparison of two classes of data. A *P* value < 0.05 was considered as statistically significant.

## Results

### CA expression in adipose tissue

**Fig. 1:**
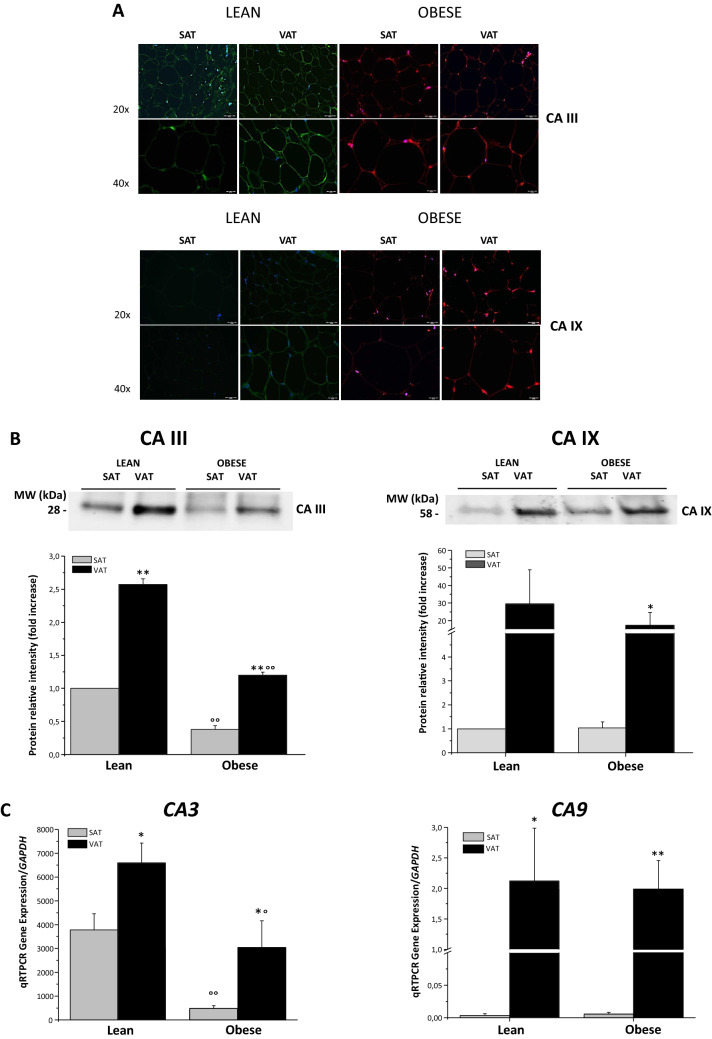
CAIII and CAIX expression in the adipose tissue. **A** Representative images of immunofluorescence staining were obtained from both SAT and VAT samples showing CAIII and CAIX expression in both lean and obese patients as red and green signals, respectively. Of note, the higher dimension of the adipocytes in AT from obese vs. lean subjects suggest pathological differences. DAPI staining (blue) was used to mark cell nuclei. Scale bar = 50 µm. **B** Western blot analysis of CAIII (left panel) and CAIX (right panel) protein expression in SAT and VAT samples from lean and obese patients. The upper panels show a representative blot for CAIII (left) and CAIX (right), while the graphs show the mean ± SE expression levels of CAs normalized to total protein loading and in fold increase vs. SAT of lean patient. *P < 0.05; **P < 0.001 SAT vs. VAT. ^°°^*P* < 0.001 lean vs. obese. **C** qRT-PCR gene expression of *CA3* (left panel) and *CA9* (right panel) normalized on *GAPDH* as the reference gene. Data are expressed as the mean ± SE performed in triplicates in at least two independent experiments. *P < 0.05, **P < 0.001 SAT vs. VAT,^°^*P* < 0.05, ^°°^*P* < 0.001 lean vs. obese (*n* = 4 lean and *n* = 8 obese subjects)

First, CAIII and CAIX expression was investigated in the adipose tissue derived from subcutaneous and visceral abdominal pads from a small cohort of lean (*n* = 4, BMI = 22.3 ± 1.4) and morbid obese (*n* = 8, BMI = 46.8 ± 14.6) subjects. Immunofluorescence analysis of paired biopsies from the same subject showed a marked positivity for CAIII at the cytoplasmatic rime in VAT adipocytes, compared to the lower signal in SAT adipocytes of a lean subject (Fig. 1A, left upper panels). A positive signal for CAIII was evident in VAT but not in SAT paired biopsies of a morbid obese subject (Fig. 1A, right upper panels). Only adipocytes in VAT displayed a relevant CAIX positivity with no substantial difference between fat from obese (Fig. 1A, right lower panels) and lean (Fig. 1A, left lower panels) subjects.

Western blot analysis for CAIII and CAIX protein confirmed the specificity of the immunofluorescence results. A specific band for CAIII displayed a higher intensity in VAT vs. SAT, and was much more evident in lean compared to obese subjects (Fig. 1B, left panels). Similarly, a specific band corresponding to CAIX protein was highly expressed in visceral vs. subcutaneous fat pads, with no substantial difference in obese condition (Fig. 1B, right panels). qRT-PCR analysis further confirmed the lower expression of *CA3* in obesity (Fig. 1C, left panel), while *CA9* expression was substantially similar in VAT and limited in SAT, independently from the metabolic conditions (Fig. 1C, right panel).

### CA expression in adrenocortical carcinoma

**Fig. 2:**
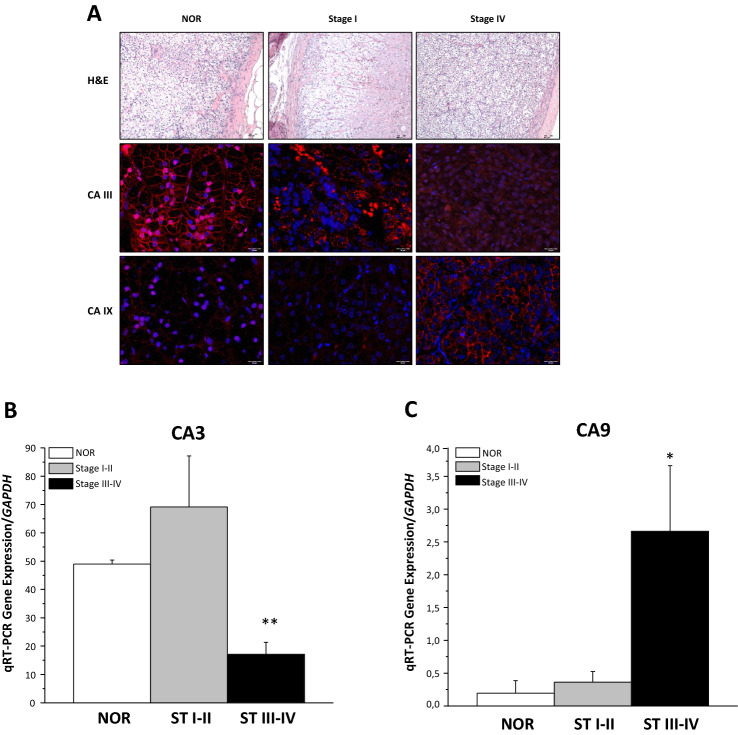
CAIII and CAIX expression in ACC samples. **A** Representative images of both hematoxylin/eosin (upper panel) and immunofluorescence (middle and bottom panels) staining were obtained in serial slices of healthy adrenal specimens (NOR), stage I ACC and advanced stage IV ACC. The expression of CAIII and CAIX is shown in adrenal cortex area as a red signal. DAPI was used for nuclear staining. Scale bar = 100 µm, 20 µm. **B** qRT-PCR gene expression of *CA3* (left panel) and *CA9* (right panel) normalized on GAPDH as the reference gene in *n* = 23 ACC and *n* = 2 healthy adrenal specimens (NOR). Data are expressed as mean ± SE performed in triplicates in at least two independent experiments. *P < 0.05; **P < 0.001 stage I–II (*n* = 9) vs. stage III–IV (*n* = 14)

To investigate the expression and the possible role of CAIII and CAIX in adrenocortical cancer, we explored expression of the two CAs in normal adrenocortical tissue compared to adrenocortical cancer stratified in low (I–II) and advanced (III–IV) stages. Immunofluorescence analysis of formalin-fixed paraffin-embedded specimens of normal adrenal was compared with representative ACCs (stage I and stage IV) (Fig. 2A). The marked positivity for CAIII protein was evident in the zona fasciculata of normal adrenocortex, and progressively reduced in stage I and in particular in stage IV ACC specimens (Fig. 2A). Conversely, CAIX was clearly present in stage IV specimens, with no evident positivity in ACC at less aggressive stage I or in normal cortex (Fig. 2A). qRT-PCR performed in *n* = 23 ACC samples (patients’ characteristics in Table 1) showed a higher expression of *CA9* in more advanced tumors (*n* = 14 ACC stage III–IV) than in lower stage samples (*n* = 9 ACC stage I–II), while *CA3* showed an opposite trend, with a significantly higher gene expression in stage I–II vs. more advanced stages. In normal adrenal specimens, *CA9* is almost undetectable, while *CA3* expression is comparable to stage I–II ACC (Figs. 2B,C).

### CAIII and IX reciprocal modulation in an in vitro model of adipose and ACC cell coculture

**Fig. 3:**
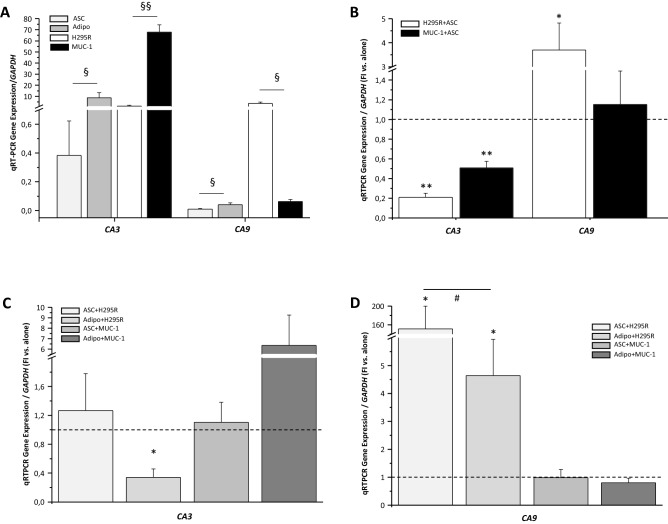
Modulation of CAIII and CAIX expression in adipose and tumor cells under coculturing conditions. **A** *CA3* and *CA9* gene expression analysis performed by qRT-PCR in adipose stem cells (ASC), in vitro-differentiated adipocytes (Adipo), H295R and MUC-1 cells. Data are expressed as mean ± SE gene expression vs. the housekeeping *GAPDH* gene. §*P* < 0.05, §§*P* < 0.001 ASC vs. Adipo and H295R vs. MUC-1. **B** qRT-PCR gene expression analysis of *CA3* and *CA9* in adrenocortical cancer cells lines in monoculture and in coculture conditions with ASCs. Data are expressed as mean ± SE gene expression normalized on *GAPDH* as the reference gene and in fold increase of coculturing conditions vs. tumor cells alone taken as 1 (dotted line). qRT-PCR gene expression analysis of *CA3* **C** and *CA9* **D** evaluated in adipose cells in monoculture and in coculture conditions with H295R or MUC-1 cells. ASCs cultured in undifferentiating conditions (ASC) or under stimulation toward adipogenesis (Adipo), alone or in the presence of tumor cells. Data are expressed as mean ± SE gene expression normalized on *GAPDH* as the reference gene and in fold increase of coculturing conditions vs. adipose cells (ASCs or adipocytes, respectively) alone taken as 1 (dotted line). *P < 0.01 cocultured conditions vs. alone; #*P* < 0.05 cocultured adipo vs. cocultured ASCs

Advanced adrenocortical cancer is characterized by disruption of the adrenal capsule enabling cancer cell infiltration of the visceral adipose pad surrounding the gland. In this condition, a crosstalk between adipose and tumor cells has previously been shown [3, 4]. Thus, we evaluated if this interaction may also exert any effect on CAIII and CAIX expression in both cell compartments, by assessing the reciprocal modulation of the two CAs in an in vitro coculture system set up between ASCs and the two adrenocortical cancer cell lines, H295R and MUC-1 cells. qRT-PCR revealed that in vitro differentiation of ASCs toward mature adipocytes was associated with a significant increase in *CA3* expression when compared to undifferentiated ASCs (Fig. 3A), while *CA9* expression was modest both in ASCs and mature adipocytes (Fig. 3A). *CA3* and *CA9* expression was significantly different between the two ACC cell lines, being the former higher in MUC-1 compared to H295R cells, with an opposite trend for *CA9* (Fig. 3A). Coculturing with ASCs resulted in a significant decrease in *CA3* in both cancer cells, to a higher extent in H295R (Fig. 3B). Conversely, *CA9* was upregulated in H295R when cocultured with ASCs, with no alteration in MUC-1 cells (Fig. 3B). When we looked at the reciprocal effect exerted by the tumor coculture on the adipose cells, no significant effect was evident on cocultured ASCs, either in the presence of H295R or MUC-1 (Fig. 3C). When ASCs were stimulated to differentiate toward adipocytes, a significant reduction in *CA3* in the presence of H295R cells and a slight increase in the presence of MUC-1 was evident compared to adipogenesis stimulated without tumor cells (Fig. 3C). The presence of H295R in coculture was associated with a *CA9* upregulation in ASC and induced adipocytes compared to monocultures (Fig. 3D); and this upregulation induced by H295R coculturing was weaker in differentiating adipocytes compared to ASCs (Fig. 3D). When coculture was conduced with MUC-1, conversely, there was no inhibition of *CA9* expression but coculture conditions resulted in a slight reduction in both ASCs and in differentiating cells, compared to monoculture conditions (Fig. 3D). When we look at the effect of coculturing adipose cells induced to differentiate in the presence of the two ACC cell lines compared to cells differentiated alone, H295R significantly inhibited adipogenesis, as assessed by qRT-PCR expression of two late genes selectively expressed by mature adipocytes (*AdipoQ*: 90% and *FABP4*: 91% inhibition), while MUC1 resulted in only a slight reduction in adipogenesis (*AdipoQ*: 22%; *FABP4*: 17% inhibition).

## Discussion

In the first part of our study, we showed that CAIII and CAIX modulation is associated with the metabolic alterations of the adipose tissue characterizing obesity, in particular in the visceral compartment. In the second part, we demonstrated a different distribution of the two enzymes also in ACC, which is further associated with the tumor aggressiveness. Moreover, using an in vitro dynamic coculturing system between adipose precursors and the two adrenocortical cancer cell lines, H295R and MUC-1 [14], we further elucidated a reciprocal modulation of these two enzymes in the crosstalk occurring between ACC and its visceral adipose microenvironment, being the altered expression of CAIII and IX occurring in the adipose microenvironment similar to what we found in VAT in obesity.

Our findings showed that CAIII expression is reduced in obesity compared to healthy conditions. CAIII is regulated in tissue response to metabolic alterations under the control of insulin. CAIII decreases in the AT of obese Zucker rats and in ob/ob mice compared to wild-type rodents [5, 6]. VAT depots are more prone than SAT to accumulate the excess of lipid and more susceptible to the metabolic derangement characterizing obesity, including the reduced sensitivity to insulin activity [19], which can justify the lower expression of CAIII we found in obese patients often suffering from insulin resistance. CAIII is also actively involved in the process of adipogenesis [7], in line with the upregulated expression we found in in vitro-differentiated adipocytes compared to their undifferentiated ASCs. In the early phases of differentiation, however, a downregulation of CAIII enables the expression of the early gene *PPARγ2* mastering the first step of adipogenesis [7]. Indeed, the reduced level of CAIII observed in visceral AT in obesity, and after adipogenesis induced in vitro in the presence of ACC cells, may suggest that a block in early phases of adipogenesis and in CAIII activity may characterize the metabolic alterations shared by the adipose cells in obesity and in ACC microenvironment.

During the processes of lipogenesis, lipolysis, and fatty acid oxidation characterizing the mature adipocytes, cells generate high levels of ROS and lipid peroxidation products, thus requiring protection through elevated activity of CAIII. CAIII upregulation seems to be disrupted in VAT of obese subjects, where we found lower expression of the enzyme, resulting in an exacerbation of the local hypoxic and oxidizing conditions associated with adipose dysfunctions. A similar dysregulation may also be required in the adipose cells interacting with ACC, to increase the oxidizing and low pH state that characterize the tumor microenvironment. Here, we demonstrated the higher expression of CAIII in ACC, which was not present in normal adrenals. Interestingly, CAIII appeared to be expressed at higher level in advanced compared to low stages of ACC, suggesting a role of this enzyme in supporting ACC aggressiveness, as already described for oral squamous carcinoma [20], but not in hepatocarcinoma [21–23]. The high metabolic rate of cancer cells is associated with an increased production of ROS that is further sustained by deregulation of cellular antioxidant systems. The derived oxidative stress concurs to support all processes underlying tumor progression, such as tumor proliferation and invasiveness, angiogenesis, epithelial-to-mesenchymal transition [24].

Our paper demonstrated for the first time the expression of CAIX in AT, where it is confined to the visceral depots and is almost undetectable in subcutaneous fat. A role of CAIX may be, thus, hypothesized in the elevated metabolic activity characterizing VAT. The increased expression of CAIX we found during adipogenesis may be necessary to counteract the intracellular pH acidification occurring during the intense lipolysis and fatty acid oxidation characterizing the mature adipocytes compared to ASCs. In line with other tumors, no CAIX is detectable in normal adrenal cortex tissue, while an increasing expression is observed in ACC from stage I to more advanced stages (III and IV), confirming CAIX as an independent negative predictor of disease-free survival in ACC [8]. The high levels of CAIX we found in advanced ACC and adipose cells in coculture contribute together with lactate export [3] to extracellular acidification characterizing the tumor microenvironment. CAIX expression and low pH in the tumor microenvironment positively correlate with tumor progression and chemoresistance [11]. Of note, elevated levels of CAIX were found in H295R cells, confirming previous results [8], while we found very low levels of this enzyme in MUC-1. This unexpected result is, however, in line with what was already found when comparing MUC-1 and H295R expression of markers of aggressiveness [25], which suggests that the latter represent an already metastasized model with a different metabolic behavior compared with the aggressive stage of H295R cells.

Advanced ACC (stage III and IV) is characterized by the disruption of the adrenal capsule [13], enabling infiltrating cancer cells to be in close contact with the visceral adipose compartment surrounding the gland. Using a coculture in vitro system set up between ASCs and two different human ACC cell lines, H295R and MUC-1, we reproduced this condition in vitro to study the reciprocal modulation of CAIII and CAIX in cancer and adipose cells, mimicking the adipose tumor microenvironment. Our study demonstrated that coculturing conditions with visceral ASCs, previously shown to increase invasiveness and proliferating ACC properties [3], resulted in CAIII downregulation and CAIX upregulation to a higher extent in H295R than in MUC-1. The reduced expression of CAIII in coculture may be necessary to maintain the elevated ROS production which supports the more aggressive and invasive phenotype stimulated by the adipose microenvironment in the primary tumor cells. At difference, MUC-1 derived from an ACC metastasis [14] may not be susceptible to this regulation that might be peculiar of the primary ACC site. A big difference in the basal level of both CAIII and CAIX expression was evident in these two cell lines, where the higher level of CAIII present in MUC-1 in basal condition may be required by the extremely high levels of ROS produced in metastatic cells. A different metabolic and steroidogenic activity as well as resistance to cytotoxicity of anticancer drugs between H295R and MUC-1 has previously been described [25–27]. A dramatic decrease in CAIII and an opposite increase were evident when ASCs were induced to differentiate in vitro toward mature adipocytes in the presence of H295R or MUC-1, respectively. The downregulation of CAIII was consistent with the ability of H295R to drastically inhibit adipogenesis and reshape the adipose precursors toward a cancer-associated adipose phenotype [3]. Indeed, less mature pre-adipocytes are characterized by lower levels of CAIII [7]. Conversely, we showed a different effect of MUC-1 on adipogenesis, which resulted to be slightly inhibited when stimulated in the presence of this ACC cell line compared to the strong block of adiponectin and FABP4 expression observed in the presence of H295R. In line with this result, CAIII slightly increased instead of decreasing in ASCs undifferentiated or stimulated to differentiate when cocultured with MUC-1. Our findings suggest the occurrence of a reciprocal modulation of CAIII between adrenal cancer and adipose cells in conditions that sustain cancer progression only in primary cancer but not in metastasis. The difference in steroidogenic ability between MUC-1 and H295R does not likely seem to underlie the differences observed in CAIII and CAIX modulation in the two cell types, as no significant differences in the levels of two enzymes were found between functional and non-functional ACCs.

The concomitant increase in CAIX in both ASCs and H295R adrenocortical cancer cells following coculture suggests a protective effect of the enzyme toward the intracellular acidification characterizing the elevated metabolic fluxes. The increase was also maintained when adipogenesis was induced in the presence of H295R. Conversely, coculturing with MUC-1 resulted in a slight reduction in CAIX expression in undifferentiated ASCs and following stimulated adipogenesis. Of note, the different response observed in the coculturing system may also be related to the nature of the ASCs in coculture. Indeed, among skin tumors, melanoma has been demonstrated to be differently modulated by subcutaneous rather than visceral fat, being the former able to stimulate invasiveness and anchorage-independent proliferation, while the latter improves cell adhesion to the matrix [28].

In conclusion, our findings highlight a modulation of CA III and CA IX expression in the crosstalk occurring between ACC and adipose stem cells, which might represent a putative target for the development of novel anticancer therapies directed against ACC adipose tumor microenvironment.
